# Silver/gold nanoalloy implant coatings with antibiofilm activity *via* pH-triggered silver ion release†

**DOI:** 10.1039/d4cc01168f

**Published:** 2024-06-25

**Authors:** Felix J. Geissel, Varvara Platania, Vasiliki Tsikourkitoudi, Justina Venckute Larsson, Thomas Thersleff, Maria Chatzinikolaidou, Georgios A. Sotiriou

**Affiliations:** a Department of Microbiology, Tumor and Cell Biology, Karolinska Institutet SE-17177 Stockholm Sweden georgios.sotiriou@ki.se; b Department of Materials Science and Engineering, University of Crete Heraklion Greece; c Department of Materials and Environmental Chemistry, Stockholm University SE-106 91 Stockholm Sweden; d Institute of Electronic Structure and Laser (IESL), Foundation for Research and Technology Hellas (FORTH) Heraklion Greece; e 3D-EM Facility, Department of Cell and Molecular Biology, Karolinska Institutet, 17177 Stockholm Sweden

## Abstract

Implant infections are a major challenge for the healthcare system. Biofilm formation and increasing antibiotic resistance of common bacteria cause implant infections, leading to an urgent need for alternative antibacterial agents. In this study, the antibiofilm behaviour of a coating consisting of a silver (Ag)/gold (Au) nanoalloy is investigated. This alloy is crucial to reduce uncontrolled potentially toxic Ag^+^ ion release. In neutral pH environments this release is minimal, but the Ag^+^ ion release increases in acidic microenvironments caused by bacterial biofilms. We perform a detailed physicochemical characterization of the nanoalloys and compare their Ag^+^ ion release with that of pure Ag nanoparticles. Despite a lower released Ag^+^ ion concentration at pH 7.4, the antibiofilm activity against *Escherichia coli* (a bacterium known to produce acidic pH environments) is comparable to a pure nanosilver sample with a similar Ag-content. Finally, biocompatibility studies with mouse pre-osteoblasts reveal a decreased cytotoxicity for the alloy coatings and nanoparticles.

## Abstract 2

AgAu nanoalloy particles deposited on Ti substrates release antimicrobial Ag^+^ ions in the presence of an acidic biofilm microenvironment, providing an on-demand triggered antibiofilm activity.

## Graphical abstract

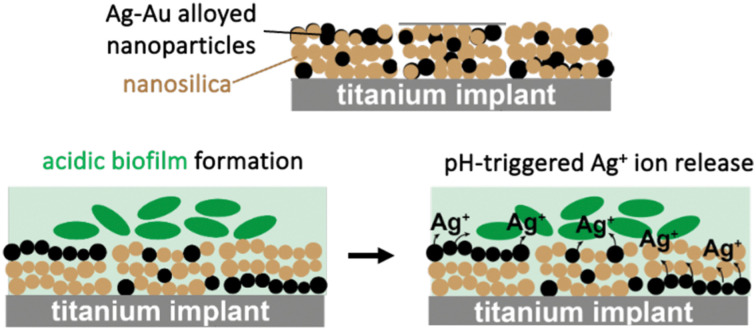

Implant infections are responsible for a high number of revision surgeries each year.^1–4^ Although surgeries are usually performed under sterile conditions, bacteria from the skin of the healthcare personnel or from contaminated medical devices can enter the body of the patient through the incision of the skin and colonize the implant. After reaching this abiotic surface, the bacteria start to form a biofilm by secreting carbohydrates and extracellular DNA, and the implant gets infected.^5^ Through this shielding nature, the microbes in the biofilm are very well protected from the only available treatment with antibiotics. The consequence is an overuse of these drugs leading to an increasing antimicrobial resistance.^6^ These two factors demand an alternative approach to keep implants contamination free and avoid costly and inconvenient revision surgeries in which the implant needs to be taken out and replaced.^7^

In the literature, surface modification of the implant is carried out by coating it with an antibacterial agent. In particular, metals and their oxides are promising due to their well-known antibacterial efficiency. Some examples are silver (Ag), zinc, copper, iron oxide and many more.^8–10^ Among those, Ag is arguably the most exploited metal due to its excellent anti-bacterial effect.^11^ The major driving force here arises from its released ions^12–16^ upon contact with fluids. This mechanism is promoted by an oxidized surface dissolving and releasing those ions.^15–20^

Several antibacterial mechanisms have been reported including hindering of DNA reproduction, preventing of protein synthesis, damaging the cell envelope and lysing the bacterial cell. While these results are very promising, Ag-containing nanostructured coatings, instead of free particles, are being investigated for their potential cytotoxicity towards mammalian cells *via* their Ag^+^ ions.^8,21–23^ These ions are crucial in overcoming biofilms, but they might also cause damage to the surrounding tissue. Hence, they should only be released in the presence of biofilm infections to minimize uncontrolled exposure to healthy tissue. This common limitation of the uncontrolled release of any antimicrobial agent into the body remains a challenge.^24^

In this study, we investigate a nanostructured coating consisting of a silver (Ag)/gold (Au) nanoalloy. Flame spray pyrolysis (FSP) is exploited to synthesise these alloyed nanoparticles as a scalable and reproducible manufacturing route and deposit them on medical grade titanium (Ti) substrates. This alloy is crucial to prevent uncontrolled potentially toxic Ag^+^ ion release in neutral pH and physiological conditions. In neutral pH environments (contact with mammalian cells only) this release is minimal, while the Ag^+^ ion release increases in acidic microenvironments caused by many bacteria^25^*via* leaching of the alloy. For this purpose, the antibiofilm activity of *Escherichia coli* (*E. coli*), a bacterium that is known to cause an acidic microenvironment and to colonize implants causing infections, is evaluated.^25^ The antibiofilm performance of the nanoalloy coating is directly compared to a pure nanosilver coating^8,19^ consisting of a similar Ag-content (20 wt%) to ensure that the total Ag present in the system is at similar levels. We further evaluate if the more inert behaviour in neutral pH conditions of the alloy coating results in a lower cytotoxicity towards mammalian cells.

Fig. 1a shows a schematic of the flame aerosol nanoparticle deposition on Ti substrates, with which a liquid precursor solution with nominal Ag content 50 at% (and 50 at% Au) is combusted resulting in the formation of the nanoparticles that are deposited on water-cooled substrates by thermophoresis.^26^ The AgAu nanoalloy particles are supported on amorphous nanostructured SiO_2_. The role of the amorphous SiO_2_ here is dual: it controls the growth of the AgAu nanoparticles and facilitates their dispersion in aqueous solutions due to its hydrophilic nature.^13,19^ The XRD patterns of the AgAu/SiO_2_ nanoparticles collected as a powder further downstream (black line) and as-deposited on a Si substrate (red line) are shown in Fig. 1b. Since the lattice parameters of Ag and Au are very similar^19^ there is no peak shift compared to a diffractogram of a nanosilver sample. However, the average crystal size *d*_XRD_ can be calculated at 5 nm. Fig. 1c shows a transmission electron microscopy (TEM) image of the nanoalloy particles in their nanoaggregate state, characteristic for nanostructured particles synthesized by FSP.^13,14^ The nanoalloy particles appear dark in that TEM image and the amorphous SiO_2_ as diffuse grey nanoparticles. The as-deposited films exhibit a homogenous and highly porous and rough morphology as shown in a top-view scanning electron microscopy (SEM) image (Fig. 1d), in line with flame aerosol deposited films in the literature with roughness ∼500 nm,^27^ while the side-view SEM reveals that the thickness is 12 μm, as shown in Fig. 1e.

**Fig. 1:**
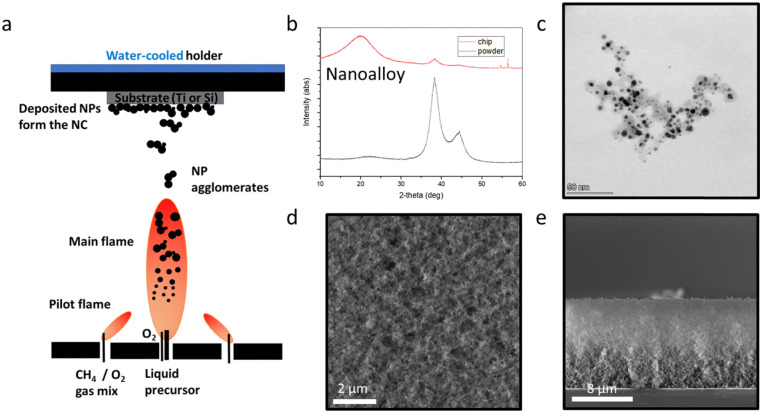
(a) Scheme of the FSP process with nanoparticles deposited on substrates by thermophoresis. (b) X-ray diffractogram of the 50 at% AgAu/SiO_2_ nanoalloy in powder form (black line) and as-deposited on the Si substrate (red line). (c) Transmission electron microscopy (TEM) image showing the alloyed nanoaggregates. (d) SEM image of the homogeneous coating surface from the top and (e) side.

To further examine the formation of a nanoalloy of Ag and Au, we perform high resolution electron microscopy analysis combining electron energy-loss spectroscopy (EELS) with energy dispersive X-ray spectroscopy (EDX). A high resolution scanning transmission electron microscopy (STEM) image of a single nanoaggregate is shown in Fig. 2a. The single Ag/Au atoms are visible and the crystallinity of the particle is highlighted. These images further suggest a single crystal phase by Ag and Au atoms together. In Fig. 2b and c, Fourier transforms of individual nanoparticles denoted with the boxed regions in 2a are presented, highlighting the crystallinity of the resulting nanoparticles. To further examine the spatial distribution of the Ag and Au atoms and validate the formation of nanoalloys, we perform elemental mapping using a recently published method called hypermodal data fusion (HyDF).^28–30^Fig. 2d shows the fraction of Ag in the alloy (Ag/(Ag + Au)) that exhibits a higher concentration in the shell of the nanoparticles. This Ag-rich layer in AgAu alloyed nanoparticles has also been validated by molecular dynamics.^31^Fig. 2e and f show the elemental distribution of Ag and Au, respectively. Both elements are homogeneously distributed throughout the particles, without any segregated Ag or Au areas. This is further shown in the composite image of Fig. 2g (see EDS spectrum in ESI†, Fig. S1).

**Fig. 2:**
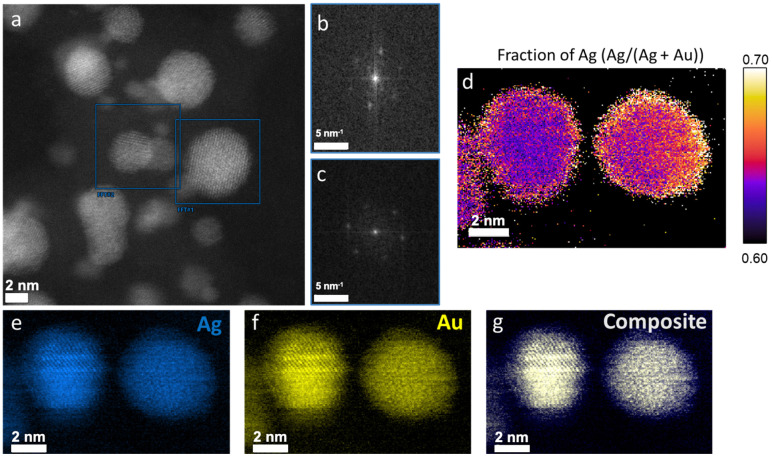
(a) High resolution TEM image of the alloyed NPs with the respective crystal phases (b) and (c). (d) Ag rich “shells” in the alloy composition. (e)–(g) Elemental mapping of Ag, Au in separate images and as a composite.

*E. coli* is a major pathogen causing many biofilm-related infections in healthcare. The clinical isolate HVM51 is grown in TSB rich medium on Ti substrates and the biofilm growth is firstly studied on the untreated control. Fig. 3a–d show SEM images of these bacteria grown on Ti substrates for 24 h. A major growth of irreversible attached bacteria is evident, which is a criterium for the biofilm definition. The high-resolution images further show flake-like structures around the bacteria, which are most likely extracellular polymeric substances (EPS) being produced in the biofilm environment.^32^ These images confirm the ability of the used strain to form a biofilm on the untreated control under the given conditions.

**Fig. 3:**
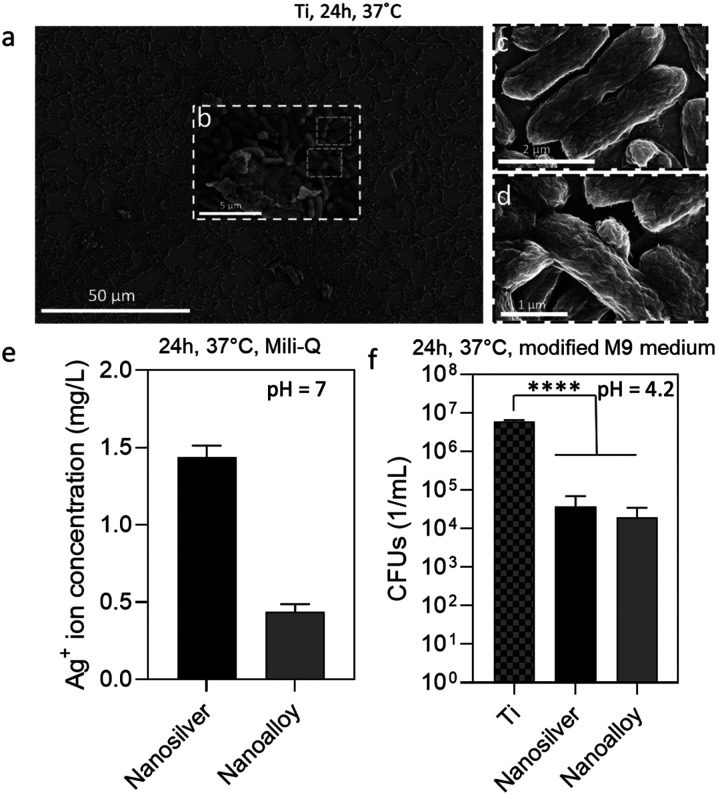
(a) SEM image of the uncoated Ti substrate after bacterial incubation. (b) Early biofilm growth with potential ECM components and (c) and (d) high resolution images of *E. coli* bacterial cell structures. (e) The Ag^+^ ion release after 24 h in ultra-pure water (physiological conditions) is significantly lower for the nanoalloy coating than for the pure nanosilver coating. (f) CFUs of uncoated Ti (control), nanosilver coating and the nanoalloy coating. There is no difference in inhibition for the two coatings indicating a similar Ag^+^ ion release only in acidic conditions. ****p < 0.0001.

To directly evaluate the effect of the Au addition on the Ag^+^ ion release from AgAu nanoalloys, we produce a pure Ag sample supported on the same nanostructured SiO_2_ at a nominal Ag-content at 20 wt%, corresponding to the same amount of Ag in the AgAu/SiO_2_ nanoparticles (50 at% Ag or 18 wt% Ag). The Ag^+^ ion release from the porous coatings is in agreement with that observed by the dispersed powders.^8,13^ The Ag average crystal size of the Ag/SiO_2_ sample is 6 nm to ensure that there are no major differences due to size effects.^8^ Upon measuring the Ag^+^ ion release of both AgAu/SiO_2_ and pure Ag/SiO_2_ nanoparticle films deposited on Ti with similar film thicknesses (16 μm ± 2 μm) incubated in pure water for 24 h at 37 °C, we observe that the nanoalloys release significantly less Ag^+^ ions than the nanosilver sample (Fig. 3e). This is because the presence of Au reduces the formation of the AgO-layer on the nanoparticle surface^33^ that is largely responsible for the Ag^+^ ion release (see release kinetics in ESI†, Fig. S2 and S3).^12,15,19^

To evaluate the pH-triggered dissolution of the nanoalloys, *E. coli* was grown in a modified minimal growth medium promoting the change towards an acidic pH environment down to pH = 4.2 after 24 h.^25^Fig. 3f shows CFUs mL^−1^ of the recovered biofilm from the untreated Ti (no Ag or Au), the nanosilver coating (Ag/SiO_2_) and the nanoalloy coating (AgAu/SiO_2_). The SiO_2_ component of the coating is not known to have any antibacterial effect.^8^ Most importantly, the biofilm inhibition for both the nanosilver coating and the nanoalloy one reaches identical levels with up to 3 log reduction (99.9%) in the CFUs mL^−1^, which is in agreement with that observed by Ag-containing coatings in the literature.^8^ The nanoalloy is as efficient as the nanosilver sample despite having a lower ion release in physiological conditions at pH 7. This suggests that the acidic pH in the biofilm microenvironment is sufficient to dissolve the Ag from both the pure Ag/SiO_2_ sample as well as the nanoalloy and to reduce the bacterial growth in a self-triggered manner (please see ESI†, Fig. S4 for the TEM image after incubation at pH 4). The flame-deposited coatings have a large porosity (up to 90%) allowing facile ion release throughout the whole film, and resembling the ion release kinetics similar to the nanopowders.

Finally, we investigated if the lower Ag^+^ ion release at physiological conditions results in a lower cytotoxicity towards mammalian cells. For this reason, the cell viability of MC3T3-E1 pre-osteoblastic cells cultured in the presence of increasing particle concentration of silica, silver and AgAu alloy nanopowders was assessed and cell morphology was evaluated *via* optical microscopy. Additionally, we analyzed the % viability on the corresponding nanocoatings. Fig. 4a shows the viability of the corresponding nanopowders. The nanoalloy powder leads to a higher cell viability at low concentrations. Fig. 4b depicts the morphology of the cells incubated with 5 and 15 μg mL^−1^ of nanopowders, as observed through optical microscopy. The cells incubated with the nanoalloy powder exhibit the characteristic elongated morphology similar to the cells in the control well (data not shown). This suggests a normal proliferation without any cytotoxic effects. However, when the cells are incubated with the nanosilver powder, they form rather spheres which can be attributed to a hindered proliferation.^34^Fig. 4c shows the % viability of cells directly grown on the different coatings compared to Ti for 1 and 2 days. All coatings show an increased cell viability on day 2 compared to day 1. After day 1, the nanoalloy coating leads to a non-significant increase of % viability in comparison to the nanosilver coating.

**Fig. 4:**
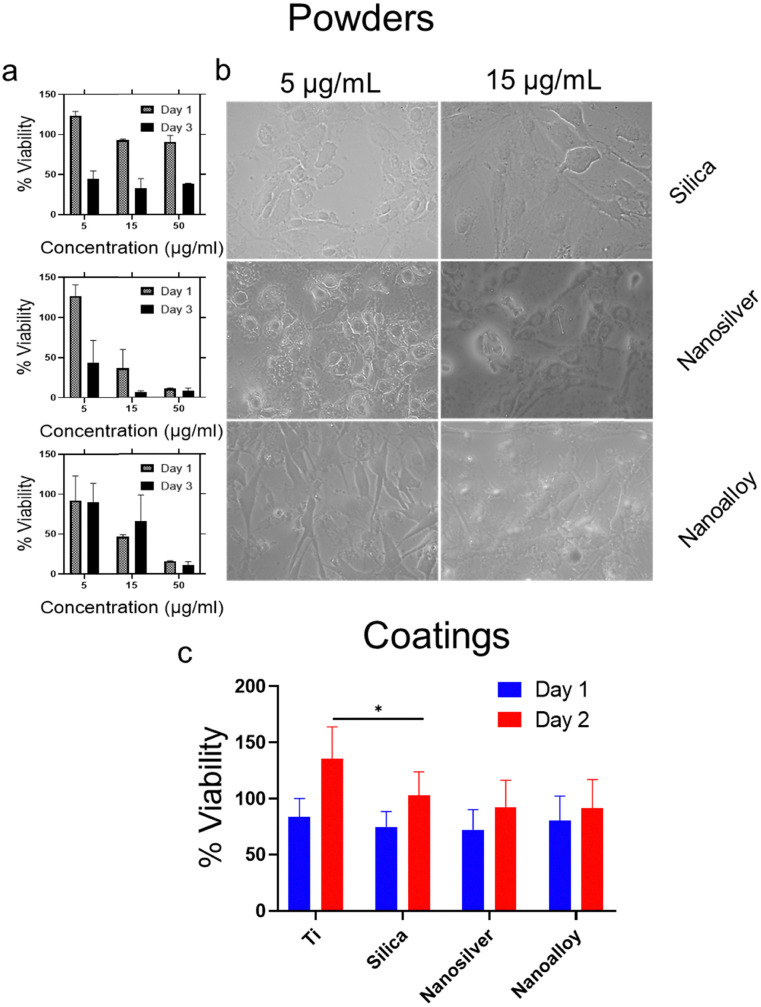
(a) % Viability of the pre-osteoblasts cultured in the presence of silica, nanosilver and nanoalloy powders and (b) corresponding cell morphology visualized by optical microscopy on day 3. (c) % Viability of cells cultured on the coatings. *p < 0.05.

This study aimed to produce an advanced implant coating with an on-demand, self-triggered Ag^+^ ion release. For this purpose, an AgAu nanoalloy was synthesized, which is more inert at physiological conditions but releases Ag^+^ ions in acidic microenvironments. The successful synthesis of this nanoalloy was achieved with FSP and, moreover, it enabled the deposition on Ti in a single step. It was also possible to reduce the release of Ag^+^ ions from this alloy in pure water when compared to a nanosilver sample with identical Ag content without affecting the antibiofilm efficiency against *E. coli*. This concludes that the nanoalloy coating is as effective in preventing biofilm formation in acidic pH microenvironments as the nanosilver sample while being potentially less toxic towards mammalian cells in physiological conditions and prompting further biocompatibility and efficacy assessment with *in vivo* models. This nanoalloy coating could help to enrich the understanding for developing a responsive implant coating with an on-demand, self-triggered Ag^+^ ion release. That would prevent unnecessary and uncontrolled distribution of Ag^+^ ions in the human body.

This work received funding from the European Research Council (ERC) under the European Union's Horizon 2020 research and innovation program (ERC Grant Agreement No. 758705). Funding from the Karolinska Institutet, the Swedish Foundation for Strategic Research (SSF) (FFL18-0043 and RMX18-0043) and the Swedish Research Council (No. 2016-05113, 2021-05494, 2021-02059, and 2023-03057) is kindly acknowledged. The 3D-EM facility at Karolinska Institutet is acknowledged for the TEM results. The authors thank Birgitta Henriques-Normark, Staffan Normark, and the BHN group (KI) for the insightful discussions.

## Data availability

Data for this article, including raw data, are available at Karolinska Institutet's Electronic Notebook platform at eln.ki.se.

## Conflicts of interest

There are no conflicts to declare.

## Supplementary Material

CC-060-D4CC01168F-s001
