# Supplementary material for: Silver/gold nanoalloy implant coatings with antibiofilm activity via pH-triggered silver ion release
Source: Chem Commun (Camb). 2024 Jun 25;60(60):7729–32. doi: 10.1039/d4cc01168f (PMC11265312; doi:10.1039/d4cc01168f)
Supplement: CC-060-D4CC01168F-s001 [file CC-060-D4CC01168F-s001.pdf]

Electronic Supplementary Material (ESI) for ChemComm.
This journal is © The Royal Society of Chemistry 2024

ELECTRONIC SUPPORTING INFORMATION

Silver/gold nanoalloy implant coatings with antibiofilm activity via a pH-triggered silver ion release

Felix J. Geissel[1], Varvara Platania[2], Vasiliki Tsikourkitoudi[1], Justina Venckute Larsson[1], Thomas Thersleff[3], Maria Chatzinikolaidou[2,4], Georgios A. Sotiriou[1,*]

[1]Department of Microbiology, Tumor and Cell Biology, Karolinska Institutet, Stockholm, Sweden.
[2]Department of Materials Science and Technology, University of Crete, Heraklion, Greece.
[3]Department of Materials and Environmental Chemistry, Stockholm University, Sweden
[4]Institute of Electronic Structure and Laser (IESL), Foundation for Research and Technology Hellas (FORTH), Heraklion, Greece.

* corresponding author email: georgios.sotiriou@ki.se

Material & Methods

Nanoparticle coating synthesis and characterization

We deposited Au and Ag together with silica (SiO_2) nanoparticles on Ti substrates (5 mm x 5 mm, Goodfellow) and *in situ* annealed the film with an ethanol flame.[15] During this process it is also possible to collect the corresponding nanopowder for quality control measurements on a glass fiber filter (AlbetLabScience) with the aid of a vacuum pump (Busch). The liquid precursor consisted of the following complex stoichiometric concentration: one final Ag content: 20 wt%[5] (20AgSiO$_2$) and 25 at% Ag alloyed with 25 at% Au (50[AgAu]SiO$_2$ final Ag nominal wt% of the alloy 18wt %) were prepared with Ag acetate (Sigma, 99%), Au acetate (Sigma, 99%) and dissolved in 2-ethylhexanoic acid (2-EHA) (Sigma, > 99%) together with acetonitrile (Sigma, > 99.8%,). After 30 minutes, appropriate amount of silicon precursor were added (hexamethyldisiloxane, HMDSO, Sigma, > 98%).

This precursor solution was then sprayed according to the FSP process previously described.[8] The height of the substrate, dispersion rate and flow rate were adjusted to match the well studied AgSiO$_2$ sample.[5] Finally, the deposition time for the alloys was increased form 60s to 100s to match the film thickness.

For particle size characterization, transmission electron microscopy (TEM) was performed with a 120 kV LaB6 microscope Talos 120C G2 and a Ceta-D detector (Karolinska Institutet, 3D-EM facility). Moreover, an advanced energy dispersive X- ray spectroscopy (EDX) mapping analysis was carried out to ensure the formation of a nanoalloy phase (Themis Z, Thermo Fisher, Stockholm University). For both preparations appropriate amounts of nanopowders were dispersed in ethanol with a sonicator. A droplet was then put on a copper grid and dried overnight before imaging.

X-ray diffraction (XRD, Rigaku MiniFlex 600) was used to determine the crystal structure of the as deposited coatings (since Ti shows a very distinct background pattern, the nanoparticles were deposited on silicon (Si) substrates) and the corresponding powder of the 50[AgAu]SiO$_2$ sample. The film morphology was evaluated with a scanning electron microscopy (SEM, Phenom Pharos, Thermo Fisher, and Gemini Ultra 55, Zeiss). The Ag^+ ion release in water (MiliQ) was measured with a sensitive electrode (Mettler & Toledo).

Ag^+ ion release in pH buffers

0.008g of particles were added in 2ml MilliQ water, the aggregates were sonicated using a cup horn sonicator for 30 min, at high power. After that the sample was diluted in a citrate phosphate buffer with a desired pH and the final noble metal concentration of the particles was 50mg/L. The diluted sample was divided into 3 bottles and 10ml of each sample was used for the Ag^+ release measurements using the Mettler Toledo silver ion selective electrode, in each sample prior the measurement the Ion Strength Adjuster was added in the recommended amount.

Biofilm growth protocol and inhibition evaluation

E. coli (HVM52) was grown in tryptic soy broth (TSB) medium at 37 °C overnight and subsequently diluted to an optical density (OD) of 0.001 at 600 nm. This corresponds to around 8 x 10^5 bacteria. Pure Ti, a silica coated Ti and 50[AgAu]SiO$_2$ were used to establish a basic growth / inhibition understanding. The three different samples were sterilized in an oven at 210 °C (Carbolite, Gero) and placed into a 48-well plate. Finally, an established biofilm growth protocol was applied to incubate the samples together with the bacteria and to assess their inhibition potential.[5]

Evaluation of increased ion release in acidic conditions

HVM52 was grown in M9 modified minimal medium[14] at 37 °C overnight and subsequently diluted to an OD of 0.01 at 600 nm. This step is crucial for promoting the acidic microenvironments. Uncoated Ti, 20AgSiO_2 (from now on referred to as "nanosilver") and 50[AgAu]SiO_2 (from now on referred to as "nanoalloy") were sterilized (as above), placed in 48 well plates and incubated with the bacterial suspension for 24 hours.

Biocompatibility studies

Pre-osteoblastic cell culture maintenance and cell seeding

We employed murine calvaria-derived pre-osteoblastic cells MC3T3-E1, passages 12 to 14 in our study. Cells were cultured in alpha-MEM minimum essential media from (PAN Biotech, Germany), supplemented with 10% FBS, 100 units mL^{-1} of penicillin-streptomycin, and 1% amphotericin. The cell-loaded coatings and nanopowder cultures were then maintained in a humidified atmosphere in an incubator with 5% CO_2 at 37 °C.

Cell viability, proliferation and morphology assessment

We quantitatively assessed the viability of MC3T3-E1 pre-osteoblasts in direct contact with silica, silver and alloy nanopowders at increasing concentrations (5, 15, 50 µg/ml) after 1 and 3 days in culture using the resazurin-based metabolic assay PrestoBlue® (Invitrogen, USA). The cell viability on the produced nanoparticle coatings was evaluated by the same method. For each sample 10^4 cells were seeded in direct contact. At designated times of culture, we introduced the PrestoBlue® reagent which was diluted at a 1:10 ratio in culture medium. This mixture was incubated at 37 °C for 60 min before measuring the absorbance at 570 and 600 nm using a spectrophotometer (Synergy HT, Biotek, USA). All experiments were conducted in quadruplicates.

After 3 days in culture, we investigated the adhesion and morphology of pre-osteoblasts in direct contact with 5 and 15 µg/ml silica, silver and alloy nanopowders by means of an optical microscope (Carl Zeiss GmbH, Germany).

Statistical analysis

The statistical analysis was performed in GraphPad Prism 9 (La Jolla, USA) using two-way ANOVA comparing the nonocoatings to the control (pure silica). For the in vitro cytotoxicity studies, the experimental data was analyzed using two-way ANOVA followed by Dunnett's multiple comparisons test between groups. The symbols designate as follows unless stated otherwise for an individual figure: *$p < 0.05$, n.s. = statistically non-significant difference compared to the pure silica control surface.

Acknowledgements

This project has received funding from the European Research Council (ERC) under the European Union's Horizon 2020 research and innovation program (ERC Grant agreement n° 758705). Funding from the Karolinska Institutet Faculty Board, the Karolinska Institutet Strategic Research Stem Cells and Regenerative Medicine (StratRegen), the Swedish Foundation for Strategic Research (SSF) (FFL18-0043 and

RMX18-0041) and the Swedish Research Council (n° 2021-05494, 2021-02059 and 2023-03057) is kindly acknowledged. We thank Prof. Birgitta Henriques-Normark, Prof. Staffan Normark and the BHN group (KI) for the insightful discussions. T.T. acknowledges funding from the Swedish Research Council (n° 2016-05113). The Karolinska Institutet 3D-EM facility is kindly acknowledged for the acquisition of TEM and SEM images and use of their equipment.

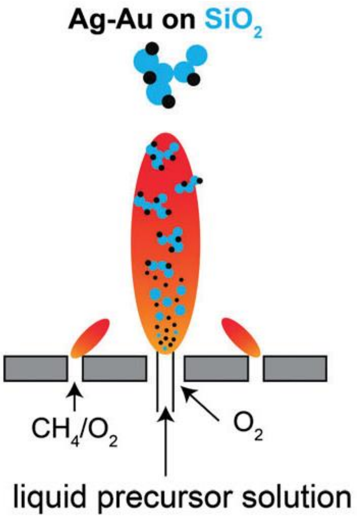

Scheme S1. Schematic representation of the flame spray pyrolysis process that yield the flame-made Ag-Au nanoparticles supported on nanostructured amorphous SiO_2.

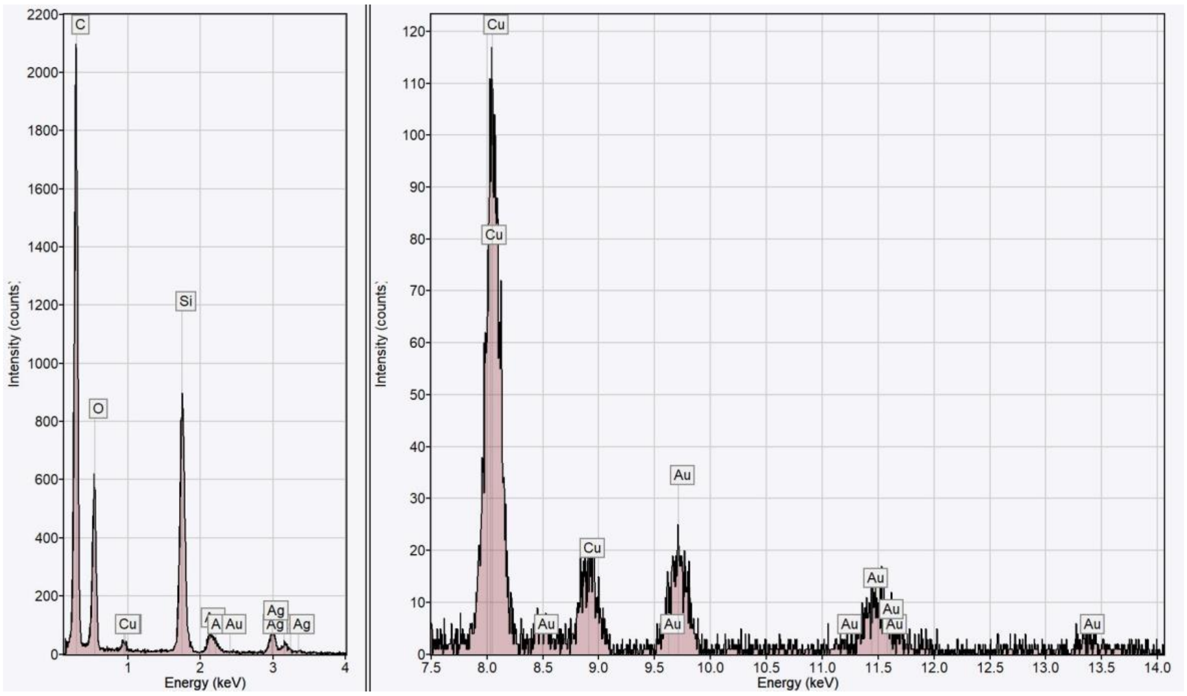

Fig. S1. The EDX spectrum of the $AgAuSiO_2$ nanoalloys that shows the peaks corresponding to O, Si, Au and Ag. The Cu and C peaks come from the carbon coated Cu TEM grid.

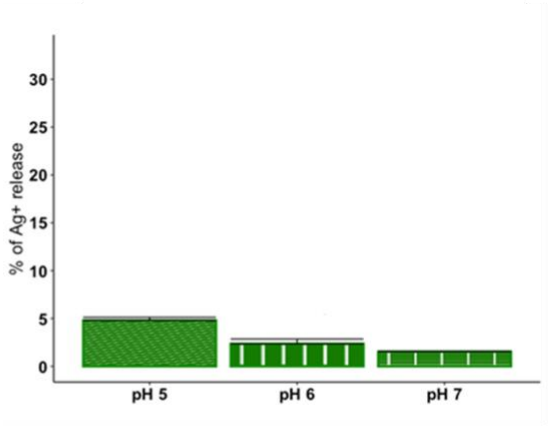

Fig. S2. Ag^+ ion release as % of the Ag-content after 48h of the nanoalloy powder in different pH buffers.

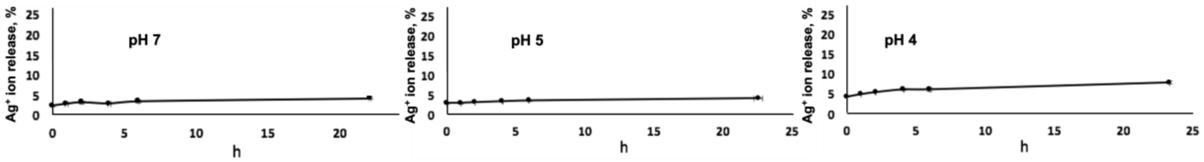

Fig. S3. The Ag^+ ion release kinetics of the $AgAuSiO_2$ nanoalloys in different pH values.

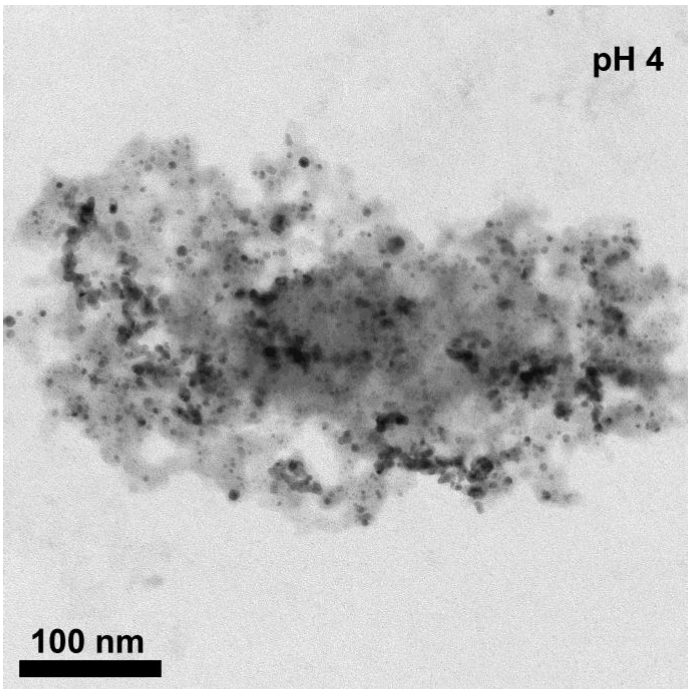

Fig. S4. Bright-field TEM image of the $AgAuSiO_2$ samples after its incubation at pH 4. There is no major morphology restructuring in the nanoparticle aggregates after incubation in acidic conditions in comparison to the pristine sample.
